# School attendance problems in adolescent with attention deficit hyperactivity disorder

**DOI:** 10.3389/fpsyg.2022.1017619

**Published:** 2022-11-23

**Authors:** Sofia Niemi, Martin Lagerström, Katarina Alanko

**Affiliations:** Faculty of Arts, Psychology and Theology, Åbo Akademi University, Turku, Finland

**Keywords:** adolescence, school attendance problems, ADHD, neurotypical, school absenteeism

## Abstract

**Introduction:** A link between having a neurodevelopmental disorder, such as attention deficit hyperactivity disorder (ADHD) and school absenteeism, has been found in previous studies. Why ADHD poses a risk for absenteeism remains unclear, and insight into the mechanisms of the association is needed. The aim of the present study was to investigate school attendance problems (SAP) and both the symptoms related and the perceived reasons for them, as reported by adolescents with ADHD (*n* = 95), compared with neurotypical adolescents (*n* = 1,474).

**Method:** The current study (*N* = 1,569) was part of the *School absence in Finland*-project. SAPs were measured with the Inventory of School Attendance Problems (ISAP). The ISAP questionnaire contains a symptom scale (ISAP S) and a function scale (ISAP F), which shows if and how the symptoms impacts school attendance. A linear mixed effects model was used to analyze outcomes on the ISAP factors, controlling for background variables living status, gender, other diagnoses, highest level of education for the parent and age.

**Results:** Results show that adolescents with ADHD had been more absent from school compared to neurotypical adolescents during the prior 12-weeks. Adolescents with ADHD showed significantly more symptoms of agoraphobia/panic, problems within the family and problems with parents than neurotypical peers. The symptoms separation anxiety, agoraphobia/panic, aggression, problems within the family and problems with parents more often were perceived as the reason for SAP (ISAP F).

**Discussion:** The results are in line with our initial hypotheses and previous studies. Because of the low response rate on the ISAP F scale, the results regarding reasons for SAPs should be interpreted with caution. Future research could examine specific preventive actions of SAPs for adolescents with ADHD, and different subtypes of ADHD.

## Introduction

School absenteeism is known to have negative consequences, as it can affect children both short term, for instance academically (Gottfried, 2009, 2014) and socially (Gottfried, 2014), and long-term causing for instance economic struggles (Ansari et al., 2020) and/or unemployment (Attwood and Croll, 2006, 2014). In the current study, the term school attendance problems (SAPs) will be used to cover all types and kinds of school absence, both legitimate/authorized and illegitimate/unauthorized. The term covers problems in all stages of the spectrum of problem severity, such as refusing or avoiding going to school, and school absenteeism. The prevalence of problematic school absenteeism in Finland among youth in secondary school is estimated to be around 2–3% (Määttä et al., 2020). The estimation was based on reports by school staff and according to the study, SAPs have increased in Finland in recent years.

There are many reasons for children to be absent from school. Health-related problems (Havik et al., 2014) and lack of good relationships with other students at school (Havik et al., 2015) are common reasons for school absenteeism. Previous studies have also shown a connection between having a neuropsychiatric diagnosis, such as autism spectrum disorder (Munkhaugen et al., 2017) and/or attention deficit hyperactivity disorder (ADHD; Kent et al., 2010; Fleming et al., 2017; May et al., 2020), and school absenteeism. There are also other risk factors, which increase the likelihood for SAPs or school absenteeism. Research shows that the risk of school absenteeism increases if a child experiences abuse, lack of care, or other kinds of problematic home conditions (Marlow and Rehman, 2021), if they come from low socioeconomic homes (Balkıs et al., 2016), or suffer from mental health problems (Egger et al., 2003). Children who display a higher level of school absenteeism during their first years in school also tend to do it later in life, meaning that the pattern of school attendance problems may be established early (Ansari and Pianta, 2019). Also, externalizing behaviors, hyperactivity, inattention, and conduct problems, are shown to be riskfactors for SAP (Ingul et al., 2011).

Attention deficit hyperactivity disorder, or ADHD, is a condition characterized by inattention and hyperactivity-impulsivity or either of them (American Psychiatric Association, 2013). To be diagnosed with ADHD the symptoms must have been present in at least two environments, for example, at school and at home (American Psychiatric Association, 2013). In addition, the symptoms must have been present for at least 6 months and have a negative impact both socially and academically (American Psychiatric Association, 2013). The symptoms must also differ from the usual level of development (American Psychiatric Association, 2013). Signs that are common in youth with ADHD are, for example, negligence at school, inability to focus on a specific thing, speaking much more than others and a habit of interrupting others (American Psychiatric Association, 2013). The prevalence of ADHD among children is between 3.4–7.2% (Polanczyk et al., 2014, 2015; Thomas et al., 2015). ADHD is more commonly found among males than among females (American Psychiatric Association, 2013).

Comorbidities are common among children with ADHD and it is, in fact, more common than uncommon to have another diagnosis or other symptoms in addition to ADHD (Biederman et al., 1991; Kadesjo and Gillberg, 2001; Yuce et al., 2013). Studies have shown that children with ADHD can have higher levels of social anxiety (Chavira et al., 2004; Schmitz et al., 2010), separation anxiety (Biederman et al., 1996), depression (Meinzer et al., 2014), agoraphobia/panic (Biederman et al., 1996, 1997), somatic complaints (Kutuk et al., 2018), and aggression (Murray et al., 2021). It is also common among youth with attention problems to have problems with peers (Barnow et al., 2006).

Children with ADHD are more absent from school compared to other children (Kent et al., 2010; Fleming et al., 2017; May et al., 2020). In addition to school absenteeism, children with ADHD also may have other kinds of school-related difficulties, for instance, low academic achievements (Fleming et al., 2017; May et al., 2020) and learning disabilities (DuPaul et al., 2012). Children with ADHD can also experience bullying more often, especially if they also have an autism spectrum disorder (ASD) diagnosis (McClemont et al., 2020). Children with ADHD are more prone to quit school earlier than others, they are more likely to need special help in school, and they are more prone to have difficulties finding a job later in life, even when the symptoms are treated with medication (Fleming et al., 2017). Furthermore, children with ADHD may have problems with emotion regulation (Graziano and Garcia, 2016). Martin (2014) found that ADHD also predicted other school-related difficulties, such as failure to complete schoolwork and needing to switch schools or being suspended from school.

As mentioned, it is common for children with ADHD to have comorbid disorders/symptoms (Biederman et al., 1991; Kadesjo and Gillberg, 2001; Yuce et al., 2013). It is important to pay attention to the comorbidities when considering SAPs, because the ADHD diagnosis alone might not be the reason for the SAP. According to Classi et al. (2012), ADHD combined with another diagnosis can increase SAPs more than ADHD alone. Their study showed that children with ADHD, who also had anxiety, depression, or phobias, were more prone to skip school for over 14 days compared to the children with ADHD only (Classi et al., 2012). This means that having ADHD and internalized problems can increase the risk of being absent from school. Another study conducted by Sciberras et al. (2014) found that children with two or more anxiety disorders in combination with ADHD had a higher degree of SAPs compared to children having ADHD and one anxiety disorder or having ADHD alone.

Having problems with peers is also common among youth with attention problems (Barnow et al., 2006), and having problems with peer relationships is also related to SAPs (Egger et al., 2003; Havik et al., 2015). As far as other relationships are concerned, children with ADHD may not have as close a relationship to their teachers as their peers do (Ewe, 2019). Also, the relationship with their parents might not be as good as compared to neurotypical children. Studies have shown that youth with ADHD have more problematic conflicts with their parents (Barkley et al., 1992; Edwards et al., 2001). The conflicts are also more aggressive, and they have a more negative tone compared to neurotypical children (Barkley et al., 1992; Edwards et al., 2001).

Concluding, prior research has highlighted several areas within education and school, which may be problematic for children and youth with ADHD. The same areas are, however, often the reason for SAP also for neurotypical children. The first aim of the present study was to compare self-reported non-attendance between adolescents with ADHD and neurotypical adolescents. The second aim related to the symptoms associated with SAP. Do neurotypical and adolescents with ADHD differ in symptom severity, and on which symptoms related to SAP that are most common?

Furthermore, it is unclear, if and to what extent the symptoms and difficulties actually contribute to or are the reason for the SAPs. The idea to differentiate between symptom and function has existed for a long time in the literature on SAP. Kearney (2008) postulated four functions of behavior: two functions relate to avoiding situations or people and two to obtaining something more desirable outside of school (activities, attention from parents). However, to the best of our knowledge, there is only one study: on the creation of the Inventory for School Attendance Problems-scale, combining the symptoms with the function of the SAP (Knollmann et al., 2018). The possibility to separate between symptom and reason is appealing, as the clinical relevance of each symptom might be different. Let us illustrate this with a hypothetical example: a young person (with or without ADHD), with a high degree of absence from school, reports about conflicts with peers and symptoms of anxiety related to test situations. Very likely both difficulties contribute to the young person feeling stressed and down. However, it might be that the reason for not attending school relates only to feeling anxious in relation to test situations. In this example, the conflicts with peers might not be perceived as a reason not to attend school, as the young person might have other friends at school, with whom he/she likes spending time. Therefore, in addition to measuring symptoms of different difficulties related to school absenteeism, it is important to also measure whether a reported symptom is also the reason (function) for not attending school. In the present study, adolescent self-reported functions for SAP are measured. However, it is important to keep in mind that perception of causes and symptoms differ between informants (Keppens et al., 2019; Knollmann et al., 2020). Parents tend to rate, e.g., anxiety higher than children/youth themselves (Knollmann et al., 2020). Also, insight into one’s wellbeing is a developing skill among adolescents. The third aim of the present study was to investigate the differences between adolescents with ADHD and neurotypical adolescents perceptions regarding functions of SAPs.

The hypotheses of the current study are:

1. Adolescents with ADHD will show a higher level of school non-attendance compared to neurotypical adolescents.
2. Adolescents with ADHD will have a higher level of the comorbid symptoms that are common among adolescents with ADHD compared to neurotypical youth: social anxiety, separation anxiety, depression, agoraphobia/panic, somatic complaints, and aggression. Adolescents with ADHD will have more problems with peers and/or teachers and/or parents.
3. Adolescents with ADHD will report that increased symptoms in the areas described in hypothesis 2 will also have an impact on their SAPs. No *a priori* hypotheses about which symptoms relate more to SAPs, or how the groups differ were made, due to the lack of previous research addressing the question.

## Materials and methods

### Procedure

The current study was a part of the *School Absence in Finland* project. The project started with translating the instruments School Refusal Assessment Scale-Revised (SRAS-R; Kearney, 2002), the Inventory of School Attendance Problems (ISAP; Knollmann et al., 2018) and the School Non-Attendance ChecKlist (SNACK; Heyne et al., 2019) into Swedish, and SNACK into Finnish. The translated ISAP questionnaire was piloted with 15 adolescents. After feedback, some smaller changes were made. Only the ISAP questionnaire and background variables were used in the current study. Voluntary schools were recruited for the study, and they recruited participants among their pupils. A total of 15 schools decided to participate in the study. The schools were located both in southern and western Finland. The data from the adolescents were collected in the school during the school day, in May 2021. Parents were contacted and informed *via* the school’s e-mail. The parents were also asked to fill out an informed consent for their adolescent below age 15 to participate in the study. The consent was collected and confirmed by the school staff at data collection. Personnel at schools participated in the data gathering process for students with a high level of school absence. Special aid teachers contacted students with high absence rates, and collected data in person, from both parents and the students.

### Ethical considerations

The study was approved by the research ethics committee of Åbo Akademi University.

### Sample

**Figure 1:**
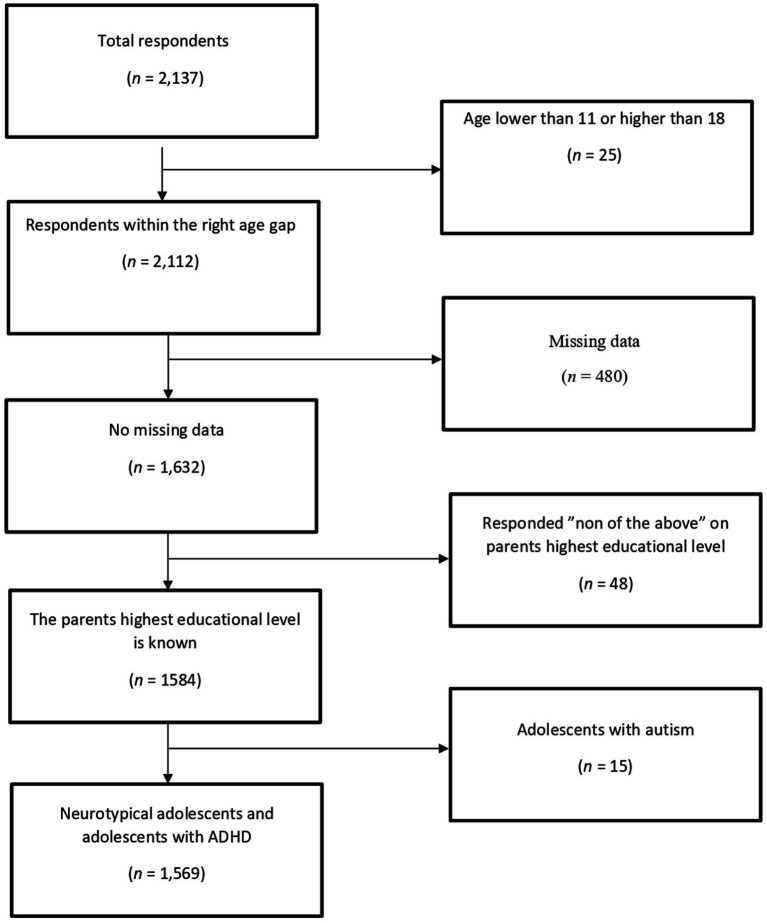
The process for the exclusion of the sample. The squares on the right side indicates the participants that were excluded from our analyses and the reason for their exclusion. The squares on the left side indicates the total sample left after the exclusion. The total sample for the study was 1,569.

The final sample with complete responses was 1,569, consisting of 952 Swedish-speaking adolescents and 617 Finnish-speaking adolescents. The average age for the neurotypical adolescents (*N* = 1,474) was 14.9 (SD = 0.85) and for the adolescents with ADHD (*N* = 95) 15.0 (SD = 1.01). The total collected sample had *N* = 2,137 responses of which 568 were incomplete and thus excluded (see Figure 1). Twenty-five participants were excluded, because they had reported an age lower than 11 or higher than 18. Four hundred and eighty participants were excluded, since they had not completed the part of the survey necessary for analyses or had more than 30% missing data. Forty-eight participants who reported “none of the above” on highest education level of a parent were excluded, since these values could not be multivariate imputed. Participants who had comorbid autism were excluded (*n* = 15), to enable comparisons between neurotypical and adolescents with ADHD.

### Measures

The questionnaire included questions on the participants’ age, gender (girl, boy, other), who the participant was living with (with both parents, with only one of them, with both alternately, or at a residential childcare community) and the socioeconomical status of the family. The socioeconomical status factor was measured by the parents’ highest educational level. The educational level was categorized into five separate groups, the highest being a university degree and the lowest to not have any type of degree after elementary school. The questionnaire also included questions about the participants other diagnoses, for example depression, cancer, asthma, and diabetes. Living status, gender, other diagnoses, highest level of education for the parent and age were included in all models, to account for variance explained by these background variables.

#### The inventory of school attendance problems

The measure for this study was The Inventory of School Attendance Problems (ISAP) questionnaire (Knollmann et al., 2018). ISAP was designed to function as a screening tool for identifying SAP at different levels of problem severity. The questionnaire contains 48 items, loading on 13 factors. The 13 factors are: problems with teachers, peers and parents, family-related problems, disapproval of the school the adolescent is in, symptoms of depression, performance anxiety, somatic complaints, aggression, social anxiety, separation anxiety, panic/agoraphobia, and having other attractive alternatives/school aversion. The ISAP questionnaire contains both a symptom scale (ISAP S) and a function scale (ISAP F). The symptom scale rates symptom severity whereas the function scale rates if and to what degree the symptom is the reason for the SAP. Both scales are answered on a 4-point Likert scale (from “0” = never to “3” = most of the time). Also, the ISAP questionnaire measures how often an adolescent has been absent from school during the last 12 weeks, both with and without permission. The following questions are examples of questions that are included in the questionnaire: “I worry that I might embarrass myself,” and “I am afraid to speak to other people or that others might speak to me” when measuring symptoms of social anxiety, and “I am afraid of exams,” and “I worry about my school grades” when measuring symptoms of performance anxiety. Internal consistency of the scale is deemed to be adequate (0.75 ≤ *α* ≤ 0.88, 3 testlets/scale; Knollmann et al., 2018). In the present sample, factor structure seems to follow the 13 factor solution reported by Knollmann et al. (2018); for more information, contact authors.

#### Data analysis

All data preparation and analyses were performed in R version 4.0.2, utilizing R-Studio version 1.3. The R package *tidyverse* (Wickham et al., 2019) was used for data handling and plotting.

Final sample size for analyses was *N* = 1,569 for ISAP S and *N* = 890 for ISAP F. Missing data was handled using the *mice* package (Van Buuren and Groothuis-Oudshoorn, 2011). For the symptom scale (ISAP S) variables, in total, 889 (57%) participants had no missing data, 567 (36%) participants had missing data on one variable and 113 (7%) participants had missing data on 2–14 variables out of 58. Of the participants, 608 (39%) had not replied to ISAP question 29 (“I am afraid of tests”). Due to the key nature of this ISAP variable for calculating the factor scores, the variable was multivariate imputed and included, despite the large number of missing values. The variable with the second most missing values was age, missing for 52 (3%) of participants. Missing values were imputed using polytomous logistic regression for highest education, gender and age and predictive mean matching for all other variables, to create a complete data set. For the function scale (ISAP F) variables, only 890 complete answers could be obtained. These were analyzed separately from the sample described above.

Linear mixed effects models were used to compare neurotypical and neuroatypical adolescents on the thirteen factors of ISAP symptoms and functions, using the *lmerTest* package (Kuznetsova et al., 2017). The school the adolescent attended was included as a random intercept, to control for variations between schools. The variance of the random effect of school was negligible, ranging from 0.00 to 0.04 (intraclass correlation, ICC: 0.00–0.07) for ISAP S and 0.00 to 0.002 (ICC: 0.00–0.01) for ISAP F. Thus, no substantial differences between schools could be found.

## Results

Background variables and frequencies are presented in Table 1. There was a larger proportion of girls in the neurotypical sample, and a larger proportion of boys in the ADHD group. Most participants lived with both parents, whereas living with one parent was more common in the ADHD group. Parent educational level did not differ between groups.

**Table 1:** Descriptive data.

Group	Neurotypical adolescents		ADHD	
	*n*	*%*	*n*	*%*
Gender				
Boy	647	44	51	54
Girl	796	54	37	39
Other	31	2	7	7
Living arrangements				
Both parents	1,143	76	53	56
One parent	129	9	22	23
Both parents alternately	186	13	17	18
Residential childcare community	8	0.5	2	2
Other	8	0.5	2	1
Parents educational level				
University	919	62	58	61
High school	515	35	32	34
Secondary school	40	3	5	5

*N* = 1,569 (neurotypical adolescents, *n* = 1,474 and adolescents with ADHD, *n* = 95). The average age for the neurotypical adolescents was 14.9 (SD = 0.85) and for the adolescents with ADHD was 15.0 (SD = 1.01).

### Comparison of symptoms and functions between groups

Independent samples t-tests were performed to compare means between the groups for both symptoms (ISAP S) and function (ISAP F) for SAPs (see Tables 2,3). The highest mean for both groups on the ISAP questionnaire measuring symptoms (ISAP S) was school aversion/having other attractive alternatives (*M* = 1.19, SD = 0.79 for the ADHD group and *M* = 0.95, SD = 0.72 for the neurotypical adolescents). The differences between adolescents with ADHD and neurotypical adolescents were significant on all the factors, except for the factor measuring performance anxiety.

**Table 2:** Means, standard deviations and differences in symptom level between adolescents with or without attention deficit hyperactivity disorder (ADHD).

ISAP factor	Neurotypical		ADHD	
	*M*	SD	*M*	SD	*t*	*p*	Cohen’s *d*
Depression	0.63	0.65	0.91	0.78	−3.5	**0.001**	0.40
Social anxiety	0.50	0.61	0.69	0.76	−2.4	**0.019**	0.27
Separation anxiety	0.31	0.46	0.43	0.54	−2.2	**0.034**	0.24
Performance anxiety	0.95	0.78	0.96	0.86	−0.18	0.855	0.02
Agoraphobia/Panic	0.21	0.43	0.46	0.62	−3.89	**0.000**	0.47
Somatic complaints	0.57	0.59	0.78	0.71	−2.75	**0.007**	0.31
School aversion/Attractive alternatives	0.95	0.72	1.19	0.79	−3.08	**0.002**	0.31
Aggression	0.64	0.68	1.08	0.87	−4.84	**0.000**	0.57
Problems with peers	0.33	0.52	0.51	0.60	−2.91	**0.000**	0.33
Problems with teachers	0.38	0.54	0.55	0.60	−2.45	**0.016**	0.28
Dislike of the specific school	0.40	0.64	0.62	0.79	−2.71	**0.008**	0.31
Problems within the family	0.29	0.57	0.59	0.81	−3.60	**0.000**	0.49
Problems with parents	0.23	0.490	0.51	0.78	−3.49	**0.001**	0.43

**Table 3:** Means, standard deviations and differences in function of symptom between adolescents with or without ADHD.

ISAP factor	Neurotypical		ADHD	
*M*	SD	*M*	SD	*t*	*p*	Cohen’s *d*
Depression	0.27	0.48	0.48	0.66	−2.16	**0.035**	0.37
Social anxiety	0.18	0.40	0.34	0.59	−1.83	0.074	0.32
Separation anxiety	0.07	0.25	0.18	0.43	−1.69	0.098	0.30
Performance anxiety	0.24	0.53	0.36	0.70	−1.12	0.269	0.19
Agoraphobia/Panic	0.09	0.28	0.26	0.51	−2.32	**0.025**	0.42
Somatic complaints	0.42	0.55	0.56	0.68	−1.41	0.166	0.23
School aversion/Attractive alternatives	0.31	0.57	0.58	0.77	−2.35	**0.023**	0.40
Aggression	0.16	0.39	0.40	0.67	−2.52	**0.015**	0.46
Problems with peers	0.14	0.36	0.26	0.56	−1.45	0.154	0.25
Problems with teachers	0.15	0.35	0.28	0.49	−1.81	0.077	0.31
Dislike of the specific school	0.13	0.38	0.27	0.60	−1.57	0.124	0.28
Problems within the family	0.11	0.38	0.32	0.63	−2.20	**0.033**	0.39
Problems with parents	0.07	0.27	0.23	0.64	−1.68	0.100	0.32

*N* = 890 (neurotypical adolescents, *n* = 843 and adolescents with ADHD, *n* = 47). Smaller sample due to the lower response rate. Significant variables highlighted in bold.

The second part of the ISAP questionnaire measured if the symptom was the reason for the participants’ SAPs (ISAP F). The highest mean for adolescents with ADHD was again school aversion/other attractive alternatives (*M* = 0.58, SD = 0.77), but the highest mean for the neurotypical group was somatic complaints (*M* = 0.42, SD = 0.55). The differences between groups were statistically significant on the factors measuring depression, agoraphobia/panic, school aversion/attractive alternatives, aggression, and problems within the family. The effect sizes for the group differences on ISAP S and ISAP F were small to moderate (Cohen’s *d*: 0.19–0.57).

### School absence, and the association between ISAP factors and school attendance problems when controlling for background variables

Sixteen percent of the adolescents with ADHD indicated that they had been absent from school at least 5–12 days during the last 12 weeks (equaling approximately 10% of school time), either with or without permission form parents and/or school. The corresponding percentage of neurotypical adolescents was 8%, meaning that the percentage of absence was twice as high among adolescents with ADHD.

Results also show that adolescents with ADHD had, compared to the neurotypical adolescents, a higher level of all the symptoms (ISAP S), except on the factor measuring performance anxiety (see Table 4). Although the adolescents with ADHD had a higher level of symptoms on most of the factors (see Table 4), the results were statistically significant on the factors measuring agoraphobia/panic (*b* = 0.16; SE = 0.05; *p* < 0.001), aggression (*b* = 0.30; SE = 0.07; *p* < 0.001), problems within the family (*b* = 0.17; SE = 0.06; *p* = 0.005), and problems with parents (*b* = 0.20; SE = 0.05; *p* < 0.001). In the multivariate analyses, living status, age, gender, other diagnoses, and the socioeconomical status were controlled for.

**Table 4:** Comparison of the symptoms (ISAP S) between adolescents with ADHD and neurotypical adolescents per ISAP Factor in multivariate analyses.

Response variable: ISAP Factor	*B*	SE	95% CI		*p*
			LL	UL	
Depression	0.09	0.06	−0.03	0.20	0.15
Social anxiety	0.05	0.06.	−0.06	0.18	0.36
Separation anxiety	0.05	0.05	−0.04	0.16	0.23
Performance anxiety	−0.12	0.08	−0.27	0.03	0.12
Agoraphobia/panic	0.16	0.05	0.07	0.25	**0.001**
Somatic complaints	0.06	0.06	−0.05	0.17	0.29
School aversion/attractive alternatives	0.12	0.08	−0.03	0.27	0.12
Aggression	0.30	0.07	0.16	0.44	**< 0.001**
Problems with peers	0.11	0.05	0.00	0.21	0.05
Problems with teachers	0.08	0.06	−0.03	0.20	0.15
Dislike of the specific school	0.12	0.07	−0.01	0.25	0.08
Problems within the family	0.17	0.06	0.05	0.28	**0.005**
Problems with parents	0.20	0.05	0.09	0.30	**< 0.001**

*N* = 1,569 (neurotypical adolescents, *n* = 1,474 and adolescents with ADHD, *n* = 95). LL = lower limits; UL = upper limits, b = neurotypical (0) vs. ADHD (1). Living status, gender, other diagnoses, highest level of education for the parent and age were included in all models, to account for variance explained by these background variables. Significant variables highlighted in bold.

Adolescents with ADHD also had higher points on every ISAP factor that showed if the symptom was the reason for their SAPs (ISAP F; see Table 5). In spite of higher points on every factor, the differences between adolescents with ADHD and the neurotypical adolescents were statistically significant only on the ISAP factors measuring separation anxiety (*b* = 0.09, SE = 0.40, *p* = 0.032), agoraphobia/panic (*b* = 0.15, SE = 0.05, *p* = 0.001), school aversion/attractive alternatives (*b* = 0.18, SE = 0.09, *p* = 0.04), aggression (*b* = 0.21, SE = 0.06, *p* < 0.001), problems within the family (*b* = 0.17, *SE* = 0.06, *p* = 0.004), and problems with parents (*b* = 0.14, SE = 0.05, *p* = 0.003) as reasons for their SAPs.

**Table 5:** Comparison of the reasons (ISAP F) for SAP between adolescents with ADHD and neurotypical adolescents per ISAP Factor in multivariate analyses.

Response variable: ISAP Factor	*b*	SE	95% CI		*p*
			LL	UL	
Depression	0.12	0.07	−0.01	0.25	0.08
Social anxiety	0.11	0.06	−0.01	0.23	0.08
Separation anxiety	0.09	0.04	0.01	0.16	**0.03**
Performance anxiety	0.06	0.08	−0.09	0.22	0.44
Agoraphobia/panic	0.15	0.05	0.06	0.24	**0.001**
Somatic complaints	0.07	0.08	−0.08	0.23	0.37
School aversion/attractive alternatives	0.18	0.09	0.01	0.35	**0.04**
Aggression	0.21	0.06	0.09	0.33	**< 0.001**
Problems with peers	0.07	0.06	−0.04	0.18	0.19
Problems with teachers	0.09	0.05	−0.02	0.19	0.10
Dislike of the specific school	0.11	0.06	−0.01	0.23	0.07
Problems within the family	0.17	0.06	0.05	0.29	**0.004**
Problems with parents	0.14	0.05	0.05	0.23	**0.003**

*N* = 890 (neurotypical adolescents, *n* = 843 and adolescents with ADHD, *n* = 47). LL = lower limits; UL = upper limits, b = neurotypical (0) vs. ADHD (1). Smaller sample due to the lower response rate. Living status, gender, other diagnoses, highest level of education for the parent and age were included in all models, to account for variance explained by these background variables.

### Clinically significant scores as reasons for SAPs

**Figure 2:**
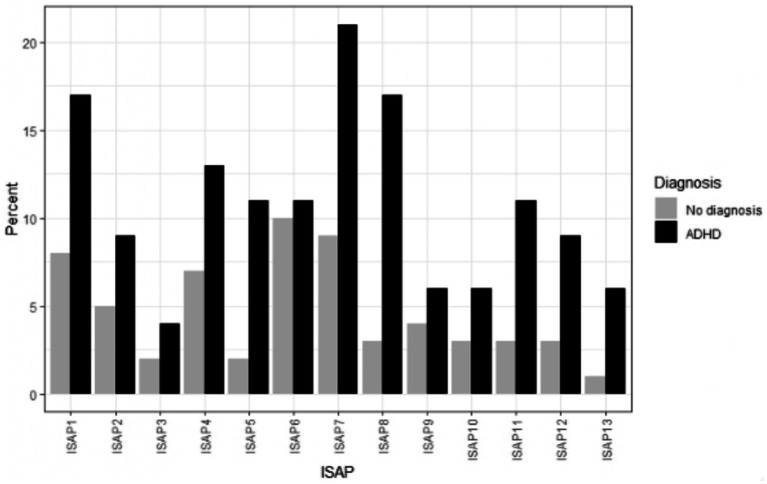
Scores indicating at least moderate influence on school attendance problems (SAP) for adolescents with and without attention deficit hyperactivity disorder (ADHD). The inventory of school attendance problems (ISAP) Function factors. The reasons for SAPs in percentages for each group per each factor in order from ISAP 1–ISAP 13: depression, social anxiety, separation anxiety, performance anxiety, agoraphobia/panic, somatic complaints, school aversion/alternatives, aggression, problems with peers, problems with teachers, dislike of the specific school, problems within the family, and problems with parents.

To further disentangle the reasons for SAPs, we analyzed scores implicating at least moderate impact of each symptom on SAPs. This was done by exploring scores above 1 (i.e., “quite often a reason”) on the ISAP F scale, in both groups (Knollmann et al., 2018). Twenty-one percent of the ADHD group had answered more than 1 on the factor measuring school aversion/other attractive alternatives (ISAP 7) as the reason for their SAPs. The corresponding percentage for the neurotypical group was 9%. School aversion/other attractive alternatives was the most common reason for SAPs among adolescents with ADHD. The most common reason for SAPs for the group with neurotypical adolescents was somatic complaints (ISAP 6), with 10%. The corresponding percentage for the group with ADHD was 11%. The least influential factor for SAPs for adolescents with ADHD was separation anxiety (ISAP 3) with 4%, and the least influential factor for the neurotypical adolescents was problems with parents (ISAP 13) with 1%. The percentage of adolescents with ADHD reporting moderate impact was twice as large compared to neurotypical adolescents on most of the factors. All factor scores above one are presented below (see Figure 2).

## Discussion

The present study aimed to investigate the differences between adolescents with ADHD and neurotypical adolescents regarding SAPs. It was hypothesized that adolescents with ADHD would have a higher level of school absenteeism compared to neurotypical adolescents. It was also hypothesized that adolescents with ADHD would have a higher level of those common ADHD and SAP-related symptoms, which were measured by the ISAP questionnaire. Furthermore, we expected that at least some of the symptoms would be perceived as the reason for the SAP. Data was gathered with the ISAP questionnaire from a total of 1,569 adolescents, aged 11–18 in different schools in Finland.

In accordance with our initial hypothesis and previous studies (Kent et al., 2010; Fleming et al., 2017; May et al., 2020), our results showed that a higher percentage of adolescents with ADHD were absent from school compared to the neurotypical adolescents. The percentage of those absent at least 5–12 days during the prior 12 weeks (equaling approximately 10% of school time) was twice as large for adolescents with ADHD (16% for ADHD and 8% for neurotypical). The cutoff we used for SAP was approximately 10% of the school time. The 10% cutoff has also been used in different contexts, for instance by the Department for Education (2019) in the UK. In our study, a significantly higher proportion of adolescents reported absence, than in the Määttä et al. (2020) study, in which Finnish professionals estimated 2–3% of middle school students were absent/had SAP. Our study likely captured emerging SAPs, compared to more severe SAPs measured by Määttä et al. Also, the period for measured school absenteeism in ISAP was relatively long, 12 weeks. Such a long time period possibly limits accurately recalling own absence (Keppens et al., 2019).

The findings show that adolescents with ADHD had a higher level of almost all the symptoms on the ISAP questionnaire. Only the factor measuring performance anxiety was lower among adolescents with ADHD, although not to a statistically significant degree. The factors measuring agoraphobia/panic, aggression and problems with parents were statistically significantly higher, when controlling also for living status, age, gender, other diagnoses, and socioeconomical status. The results are, therefore, in line with the hypothesis and in accordance with previous research showing that it is common among adolescents with ADHD to also have agoraphobia/panic (Biederman et al., 1996, 1997), aggression (Murray et al., 2021), and problems with parents (Barkley et al., 1992; Edwards et al., 2001). Even if the other factors did not reach statistical significance, it seems that adolescents with ADHD may struggle with difficulties in many areas, when comparing to neurotypical adolescents.

The adolescents with ADHD also showed higher scores on every ISAP factor showing if the symptoms were the reason for their SAPs (ISAP F). The results were statistically significant on the factors measuring separation anxiety, agoraphobia/panic, school aversion/other attractive alternatives, aggression, problems within the family, and problems with parents. The results are in line with the hypothesis, that is, the symptoms that are common among adolescents with ADHD have an impact on their school attendance. The results also support previous research about how ADHD alone might not explain the SAP and that having comorbid symptoms can increase SAPs more than ADHD alone (Classi et al., 2012). This thought is supported also by the fact that the symptoms that are typical for SAP in neurotypical youth, are even more common among adolescents with ADHD, pointing to that ADHD in itself poses a risk factor for other difficulties, which in turn may be the reason for SAP. In the present study, only some of the reasons were statistically higher in the ADHD group, however, implying that special attention should be given to these reasons. However, as a tendency for higher scores on the other reasons for SAP in the ADHD group, also these reasons should be considered when investigating the school situation for youth. Also, when a young person presents with SAPs, the investigation of reasons should also include the possibility that the challenges are due to challenges related to the neuropsychiatric condition.

Agoraphobia/panic as a reason for SAPs is not surprising considering the clinical picture of SAPs. Agoraphobia is described as having a desire to avoid situations or places that one cannot easily escape (American Psychiatric Association, 2013). There might be fear of having a panic attack at a specific place (American Psychiatric Association, 2013), in this case, is the school. Feeling a need to avoid places and situations that trigger panic is a common feature in SAP (Kearney, 2008; Heyne et al., 2019). Anxiety/panic attacks as a comorbid syndrome to ADHD may explain the higher occurrence of this problem in the ADHD group, and has also previously been reported to increase absence among children with ADHD (Classi et al., 2012).

Aggressive behavior among adolescents with ADHD has been found in prior studies. Aggressive behavior could be related to being suspended from school, and hence, also to SAP. Also, aggression could be related to problems with peers and/or teachers, even if those factors were not significantly different between the groups. School aversion/other attractive alternatives could be interpreted as truancy, i.e., absence due to low motivation, and the desire to do something more rewarding outside of school, often without the knowledge of parents and/or school (Heyne et al., 2019). In addition, school aversion could be linked to the adolescent’s inability to concentrate (American Psychiatric Association, 2013) and/or not getting the support needed in school. Insufficient support might lead to the desire to do something more enjoyable outside of school, i.e., becoming an issue of motivation. The results also showed that the most common reason for SAPs for the ADHD group was school aversion, with scores above one on the ISAP Function scale for 22% in the ADHD group (compared to 9% in the control group). A systematic review of interventions to address truancy showed, that interventions that aimed at heightening school engagement were effective in bringing students to school, in contrast to interventions, in which a punitive approach was the leading incitement (Keppens et al., 2019). Support for school engagements, especially for youth with ADHD, could be a focus for prevention of SAPs.

The significant results regarding separation anxiety were unexpected considering that children with ADHD have more problematic conflicts with their parents compared to neurotypical children (Barkley et al., 1992; Edwards et al., 2001) and that problems within the family (ISAP 12) and with parents (ISAP 13) were also significant in our study. The mean scores on separation anxiety were lower than for other factors, reflecting the adolescent developmental stage of the sample. Also, it could be speculated that adolescents with ADHD have ambivalent feelings towards their parents or that the problems between the adolescents and their parents might bring up a fear of losing them. Clearly, more research into the factors affecting SAP among neuroatypical youth is needed.

Lastly, a note on self-report data. It is important to remember that adolescents might not fully understand their symptomatology and difficulties. Adolescents can have symptoms of depression or anxiety, but they might have a hard time recognizing, and putting their feelings into words. Therefore, the self-evaluation of symptoms should be made multiple times and/or together with a close adult for an increased understanding of the symptoms. It is also important to gather information from multiple informants, such as parents and school personnel.

### Strengths and limitations

The current study comes with certain strengths and limitations. The study had 1,569 participants, and the relatively large sample size can be seen as a strength in the current study. However, the sample was not representative of the adolescent population in Finland. In addition, the ADHD group had only 95 participants, which might have led to the statistical power not being optimal, and some differences did not reach statistical significance. The ADHD group’s sample size also meant that the comparison between different subtypes of ADHD was not possible. However, the symptomatology between different subtypes may differ significantly, and future studies should analyze subgroups separately. Furthermore, the questionnaire was lengthy, possibly affecting willingness to complete it.

Another limitation is that all the participants did not answer all the questions in the questionnaire. The second part of the ISAP questionnaire (ISAP F), that is, the part that measures if the symptoms are the reason for the participants SAPs, had a low response rate with answers only from 57% of the participants. Also, the item concerning being afraid of tests was not answered by 39% of participants, reflecting possible problems with this specific item. Because of the low response rate, the results regarding reasons for SAPs should be interpreted with caution. It can be speculated that the reason for the low response rate could be due to not understanding the instructions on how to fill in the questionnaire correctly, or that the participants found it difficult to evaluate if the symptoms were the reason for their SAPs. Also, a missing answer on the function scale could be interpreted as a zero, that is, no impact on school attendance, if the participant had replied not having the symptom in question.

### Conclusion

In conclusion, the current study shows differences between adolescents with ADHD and neurotypical adolescents regarding SAPs. This study considers both symptoms that are linked to SAP and to what extent the symptoms are the reason for school attendance problems. The result of this study showed that adolescents with ADHD reported both more symptoms related to SAP, and that the symptom more often was the reason for the SAP. However, the associations reached statistical significance only for part of the symptoms and reasons. The symptoms agoraphobia/panic, aggression, and problems with parents were also perceived as reasons for SAPs. In addition, school aversion and problems with family and separation anxiety were statistically higher among adolescents with ADHD as reasons for SAPs.

Future research could examine differences between adolescents with different combinations of neuroatypicalities, such as ADHD in combination with autism spectrum disorder, and how additive diagnoses affect school attendance and possible SAPs. Future research should also examine which protective actions could be used to prevent school absenteeism in neuroatypical adolescents.

## Data availability statement

The datasets presented in this article are not readily available because the dataset is available after the end of the project period, starting 2024. Requests to access the datasets should be directed to katarina.alanko@abo.fi.

## Ethics statement

The studies involving human participants were reviewed and approved by Åbo Akademi Ethical Board. Written informed consent to participate in this study was provided by the participants' legal guardian/next of kin.

## Author contributions

KA is the principal investigator, responsible for design, data collection and revision of intellectual content. SN is responsible for drafting of the manuscript, and parts of statistical analyses. ML conducted statistical analyses and revised the manuscript for intellectual content. All authors contributed to the article and approved the submitted version.

## Funding

The study was funded by the C.G. Sundell foundation.

## Conflict of interest

The authors declare that the research was conducted in the absence of any commercial or financial relationships that could be construed as a potential conflict of interest.

## Publisher’s note

All claims expressed in this article are solely those of the authors and do not necessarily represent those of their affiliated organizations, or those of the publisher, the editors and the reviewers. Any product that may be evaluated in this article, or claim that may be made by its manufacturer, is not guaranteed or endorsed by the publisher.
